# Malignant Pleural Mesothelioma: A 2025 Update

**DOI:** 10.3390/jcm14031004

**Published:** 2025-02-05

**Authors:** Giuseppe Cardillo, David Waller, Sara Tenconi, Vincenzo Di Noia, Sara Ricciardi

**Affiliations:** 1 Unit of Thoracic Surgery, Azienda Ospedaliera San Camillo Forlanini, 00152 Rome, Italy; 2 UniCamillus, International Medical University in Rome, 00131 Rome, Italy; 3 BartsHealth NHS Trust, London EC1A 7BS, UK; david.waller1@nhs.net; 4 Manchester University NHS Foundation Trust, Manchester M13 9WL, UK; sara.tenconi@nhs.net; 5 Medical Oncology 2 Unit, IRCCS Regina Elena National Cancer Institute, 00144 Rome, Italy; vincenzopio.dinoia@ifo.it

## 1. Epidemiology and WHO and 9TH TNM Classification

The incidence of pleural mesothelioma (PM) is slightly increasing, with 2417 new cases/year worldwide. Between 2010 and 2020, stage I disease increased from 13.5% to 15.6%, and patients who were offered treatment increased from 71% to 76% [1].

According to the 5th edition of the WHO classification [2], primary malignant pleural disease can be categorized as follows:

- Localized pleural mesothelioma.
- Diffuse pleural mesothelioma.
- Epithelioid mesothelioma.
- Sarcomatoid mesothelioma.
- Biphasic mesothelioma.

In the last edition of TNM (9th TNM) have been incorporated changes proposed in the clinical (c)T but not pathological (p)T component. The importance of pleural thickness measurements has been defined, concluding that both size (sum thickness) and extent of the primary tumour (involvement of anatomic structures) are valuable in defining distinct T categories with respect to OS. Fissure involvement has been introduced in both (c)T and (p)T as important parameters, while non-transmural invasion of diaphragm (T2), lung parenchyma (T2), and endothoracic fascia (T3) have been removed as T descriptors as they cannot be accurately determined based on CT. No changes in N and M descriptors have been introduced. Stage grouping is defined as follows: stage I (T1N0), II (T1N1; T2N0), IIIA (T1N2; T2N1/2; any T3), IIIB (any T4), and IV (any M1) [3].

## 2. Principles of Mesothelioma Treatment

Multimodality therapy with systemic chemotherapy, surgical resection, and sometimes radiation therapy is generally offered to young patients with good performance status, localized disease, and epithelioid histological subtype [4,5].

### 2.1. Surgery

The definitive role of surgery in the treatment of mesothelioma remains to be determined. There is a spectrum of radicality from, at one extreme, extrapleural pneumonectomy (EPP), which is an en bloc resection of the lung, pleura, pericardium and diaphragm, through the lung-sparing pleurectomy/decortication (PD) to the most conservative, partial pleurectomy.

All current oncological guidelines emphasize the importance of multidisciplinary discussion, careful staging, including PET-CT, selection of only early-stage node-negative epithelioid disease, and the use of PD over EPP wherever possible [6,7,8,9,10].

The United Kingdom has conducted three major randomized trials in the 21st century evaluating the roles of the various surgical options:

- MesoVATS trial: An RCT comparing VATS partial pleurectomy (VATS-PP) versus talc pleurodesis (administered by thoracoscopy or chest drain-talc slurry) showed that VATS-PP did not improve patients’ survival. The authors concluded that as VATS-PP offered no survival benefit, talc pleurodesis should be preferred due to the fewer complications and the shorter hospital stay. However, there was a quality-of-life benefit in longer-term survivors due to improved effusion control from PP [10].
- MARS trial: patients were randomly assigned to chemotherapy with or without EPP. This feasibility trial did not proceed to a full phase III study due to slow recruitment and possible detrimental effects on survival from EPP compared to chemotherapy alone [11].

MARS 2 trial: The most recent RCT compared surgery + chemotherapy vs. chemotherapy alone. The results of the study showed a median survival time of 19.3 months in the surgical arm (EPD) versus 24.8 months in the non-surgical arm, with better QoL for the non-surgical arm and greater number of serious adverse events, i.e., atrial fibrillation and prolonged air leak in the surgery arm [12]. Problems with the trial design prevent its universal application, although its publication has significantly reduced the use of radical surgery. The trial was non-selective and included those with non-epithelioid, node-positive, locally advanced disease. Requiring extended surgery in elderly patients with co-morbidity resulted in excessive treatment-related morbidity and mortality and an inability to tolerate multimodality therapy.

There have been other trial programs, i.e., the SMART program in Canada, exploring the use of radical surgery (both EPP and PD) with radical radiotherapy in selected patients.

In conclusion, the favourable survival in stage I and II epithelioid mesothelioma reported in the latest 9th TNM staging edition suggests that there is a need for further research in well-staged, fitter patients. However, the relative scarcity of such cases and the numbers required to adequately power such trials will require coordinated international collaboration.

### 2.2. Radiation Therapy and Systemic Therapy

Radiation therapy can relieve symptoms in advanced disease, while its role after an incomplete surgical resection is still controversial due to its high toxicity to the intact lung [13].

Systemic therapy represents the only treatment for patients with advanced-stage disease, allowing a median survival of approximately 1 year [14].

Before the introduction of immunotherapy, the combination of a platinum analogue (cisplatin or carboplatin) with pemetrexed was considered the standard for first-line therapy in unresectable PM on the basis of the results of the phase III EMPHACIS TRIAL and data from the Expanded Access Program [15,16].

The MAPS phase 3 clinical trial has shown that the combination of Bevacizumab and a Cisplatin–Pemetrexed regimen significantly improves OS over standard chemotherapy (HR 0.77), with a median OS of 18.8 months [17]. However, this regimen is currently utilized only for selected PM patients in France.

In current clinical practice, patients with PM who maintain a good ECOG PS after failure of the first-line platinum–pemetrexed therapy can be retreated with the same front-line drugs or receive a second-line treatment with other cytotoxic drugs, such as gemcitabine and vinorelbine [18].

### 2.3. Checkpoint Inhibitors Targeting CTLA4 and PD-1/PD-L1

Checkpoint inhibitors, particularly nivolumab, have demonstrated efficacy in second-line PM treatment, especially after the failure of platinum–pemetrexed chemotherapy.

Two-phase, two single-arm trials have shown the activity of nivolumab in this setting. The NivoMes study achieved a disease control rate (DCR) of 47% at 12 weeks, with a median OS of 11.8 months [18]. The MERIT trial reported an ORR of 29%, with better outcomes linked to PD-L1 expression [19].

Moreover, the phase 3 CONFIRM trial demonstrated that nivolumab significantly improves PFS and OS over placebo in heavily pretreated patients (57% of patients were treated in the third-line setting) [20].

If PD-L1 expression (≥1%) is not predictive of either PFS or OS, statistically significant improvements in PFS and OS are reported in the subgroup analysis of patients with epithelioid histology but not in non-epithelioid patients. These data justify using an anti-PD-1 inhibitor in PM patients after failure with platinum–pemetrexed-based chemotherapy.

Pembrolizumab was first assessed in the KEYNOTE-28 trial for PD-L1-positive PM and the keynote-158 trial in patients not selected for PD-L1, showing ORRs of 20% and 8%, respectively [21,22].

However, the phase 3 PROMISE-MESO trial showed no significant improvement in both PFS and OS compared to chemotherapy despite a higher ORR with pembrolizumab (22% vs. 6%) [23].

Moreover, avelumb was evaluated in pretreated patients with PM in the JAVELIN study, with an ORR of 9% and a median OS of 10.7 months; patients with higher PD-L1 expression had better outcomes [24].

Combining checkpoint inhibitors targeting CTLA4 and PD-1/PD-L1 has shown encouraging results in refractory to first-line treatment PM. The NIBIT-MESO-1 trial demonstrated a 28% ORR with tremelimumab and durvalumab, while the INITIATE trial reported a 67% DCR with nivolumab and ipilimumab [25,26]. The MAPS2 trial highlighted the combination’s superiority over nivolumab alone, with higher ORR (28% vs. 19%) and longer PFS and OS, albeit with more grade 3–4 adverse events, which limited the widespread use of the combo in this setting [27]. However, these data encourage the evaluation of checkpoint inhibitor combinations in first-line settings.

First-line combo-immunotherapy has been established as a new standard of care by the CheckMate 743 (CM743) trial of ipilimumab and nivolumab [28]. The updated 3-year survival analysis, with a median follow-up of 43.1 months, reveals substantially improved mOS of 18.1 months in the immunotherapy group and 14.1 months for platinum-based chemotherapy [25]. Considerably, the most relevant survival benefit with the dual IC has been observed in the non-epithelioid histotype compared to that observed with chemotherapy (16.5 vs. 8.8 months; HR 0.46; 95% CI: 0.3–0.7). This result has led to the approval of the dual ICI regimen in several countries, but only in the non-epithelioid histology in Italy. An inflammatory signature score, which is based on the evaluation of RNA expression of CD-8A, PD-L1, LAG-3, and STAT1, seems to represent a potential predictive biomarker of response to ICI combination [29].

More recently, results for the IND.227 study of first-line platinum-based chemotherapy with pembrolizumab (CPP) or without pembrolizumab (CP) have revealed improved OS with a hazard ratio of 0.79 (mOS 16.13 for CP versus 17.28 for CPP, *p* = 0.0324) [30].

In addition, in the combination arm, 62% of the patients achieved an objective response. This reinforces the benefit of immunotherapy in PM and, as shown in CM743, has revealed a larger survival difference for non-epithelioid in the exploratory subgroup analysis. However, this combination has not yet been introduced in Italy.

## 3. Conclusions

In the complex scenario of a difficult disease, too many open questions are still unresolved. Despite the increasing incidence of pleural mesothelioma, our weapons to fight it are still limited: there is no universally accepted surgical therapy, and the role of immunotherapy is still debated. It is clear that future studies and RCTs are mandatory in order to establish a more accurate definition of clinical staging and appropriate characterisation of surgical criteria, as well as the benefits and toxicity of immunotherapy in PM.
